# Tracking the dropout patients of neoadjuvant chemotherapy with locally advanced oral cavity cancer

**DOI:** 10.1186/s12885-021-08420-4

**Published:** 2021-06-03

**Authors:** Jin-Ye Fu, Chen-Ping Zhang, Zhi-Yuan Zhang

**Affiliations:** Department of Oral & Maxillofacial – Head & Neck Oncology, Shanghai Ninth People’s Hospital, College of Stomatology, Shanghai Jiao Tong University School of Medicine, National Clinical Research Center for Oral Diseases, Shanghai Key Laboratory of Stomatology & Shanghai Research Institute of Stomatology, 639 Zhi Zao Ju Road, Huang Pu District, Shanghai, 200011 People’s Republic of China

**Keywords:** Drop-out patients, Neoadjuvant chemotherapy, Oral cancer, Median survival, Decision making, Nonsurgical treatment

## Abstract

**Background:**

Patients with locally advanced oral cavity cancer sometimes stopped treatment after neoadjuvant chemotherapy. There are no guidelines of the management for these patients. Before designing clinical trials, we conducted this study to investigate their characteristics, reasons of dropout, and the follow-up information.

**Methods:**

Medical records were consecutively reviewed of patients with locally advanced oral cavity cancer who underwent neoadjuvant chemotherapy from Jan 2017 to Dec 2019.Variables were compared between patients stopped treating after chemotherapy and completed treatments by student t-test and Chi-square test. Logistic regression model was used to calculate the odd rations of potential predictors of dropout. The dropout patients were followed up for reasons and results of their decision.

**Results:**

A total of 171 patients were included with 23 not undergoing surgery after chemotherapy. The odd ratios of age over 65 and single marital status were 3.11 (95%CI: 1.1, 8.7) and 4.935 (95%CI: 1.5, 16.1), respectively, for the dropout. The median survival of patients without surgery was 7.4 months. Believing that chemotherapy would be effective and being afraid of the consequence of surgery were the main reasons of refusing surgery.

**Conclusions:**

The prognosis was poor of these dropout patients. Symptom relief and fear of surgery were the reasons of dropout. Age and marital status affected their decision. Clinical trials are needed to be designed for these patients.

## Introduction

Locally advanced oral cavity cancer is highly morbid and life threatening. The conventional treatment modality is radical rection with free flap reconstruction, followed by radiotherapy or chemoradiotherapy [1]. However, the probability of cure is still low [2, 3]. Neoadjuvant chemotherapy, followed by radical surgery, may be a choice for these patients. Its advantages include downsizing the primary tumor to improve locoregional control and reducing the incidence of distant metastasis by targeting circulating tumor cells. TPF regimen, that is taxane/ platinum/ 5-fluorouracil triplets, has obtained promising results [4–8]. Thus, this strategy has been applied in clinical practice for selected patients with locally advanced oral cavity cancer in our department.

Along with the application of neoadjuvant chemotherapy, there has created a population of dropout patients, who refused to follow the protocol of undergoing surgery after taking the medicine. They had initially planned to treat the disease with radical curative management but did not adhere to the procedure after chemotherapy. There were no guidelines on how to treat this group of patients. In addition, because of drop out, their follow-up information was usually ignored. The reasons and results of these patients have little been reported in literatures. Understanding why they did not maintain in treatment and showing their follow up information would improve the clinical work of treating oral cavity cancer patients. And, for the planning of clinical trials, this entity may be a unique one to study for.

In this study, we focused on the population who withdraw from neoadjuvant chemotherapy in treatment for locally advanced oral cavity cancer and exhibited the patients’ follow up information, including the reasons and results of their decision.

## Materials and methods

### Ethics

The ethical approval was waived by the institutional review board of our hospital in view of the retrospective nature of the study and all the procedures being performed were in conformity with the provisions of the Declaration of Helsinki.

### Data acquisition

We consecutively reviewed medical documents of patients with locally advanced oral cavity cancer who had undergone neoadjuvant chemotherapy of TPF regimen in our department from Jan 2017 to Dec 2019. The inclusion criteria were patients with newly diagnosed and histopathologically proved squamous cell carcinoma of oral cavity, which is of tongue, buccal, gingiva, palate, and floor of mouth. The tumor stages were clinically classified as T2 to T4a with N1 or N2, or clinical stage of N0 with T3 or T4a, according to the Cancer Staging Manual of American Joint Committee on Cancer [9]. Radical surgery was planned to be performed after neoadjuvant chemotherapy at the beginning of treatment. The treatment plan was recorded in medical history document. The exclusion criteria were patients with distant metastasis, serious concomitant diseases, or the follow up information was not available. Patients who refused to have radical surgery before chemotherapy were not included in this study.

The retrieved information included the demographic characteristics, the chemotherapy regimen, response and adverse of chemotherapy, and the surgical performance. Patients without surgical records were followed up by telephone to themselves. If there was no answer from the patients, we called the patients’ immediate families, namely parents, children, or spouse, who lived together with the patients and took care of them during the treatment. The phone calls first confirmed whether the surgery had been done. If it was confirmed that the patients had not undergone surgery, open questions would be asked, including reasons of absence of surgery, treatment after neoadjuvant chemotherapy, reasons of taking such management, changes of patients’ symptoms, and the final results of the patients. The persons who answered the phone were encouraged to describe as detailed as possible. The relationship of the person who answered the phone with the patient and the date of the call were recorded. The data in the study was derived from these telephone communications, including ascertaining the patient’s reasons for withdrawn and changes of symptoms.

Definitions of response to chemotherapy were conformed to the Response Evaluation Criteria in Solid Tumors version 1.1 [10]. In brief, tumor response to chemotherapy was evaluated during day 21 to day 28 after first dose given. The comparison was done by physical examination and radiological images. Tumor response to chemotherapy was classified as CR (complete remission, complete disappearance of all tumor lesions), PR (partial response, tumor residual with reduction of the largest dimension ≥30%), PD (progressive disease, enlargement of tumor size ≥20% or new tumor lesion manifestation), and SD (stable disease, tumor dimension changes between PR and PD and no new lesions).

Adverse events of TPF regimen were recorded of reduced white blood cell counting and frequency of vomiting in medical records. They were graded according to National Cancer Institute Common Terminology Criteria for Adverse Events version 5.0 [11].

### Statistical analysis

Data analyses were performed using SPSS version 19.0 for Windows (SPSS Inc., Chicago, IL). For continuous variables, the mean, median, and standard deviation were calculated. Survival time in months for dropout patients was measured from the date of first dose of chemotherapy given to the date of death. Variables of smoking and drinking habits were only compared in men patients, because few women patients reported having these habits. Independent-sample t-test was used for continuous data comparison. Chi-squared test or Fisher’s exact test were used for comparisons of categorical data between patients with and without surgery. *P*-values were two-sided and smaller than 0.05 were considered statistically significant.

Multivariable analysis was done by logistic regression model to obtain odds ratios (OR) and 95% confidence interval (95%CI) of the baseline characters for dropout group compared to treatment completion group. Factors included in the regression model as potential predictors were sex (men, women), age (≤65, >65 years), marital status (married, never married/divorced/widowed), educational level (college and above, under college), and tumor stage (Stage III, IV).

## Results

### Baseline characteristics of patients

A total of 171 patients were eligible during the study period, including 148 undergoing surgery and 23 without surgery after chemotherapy. The baseline characteristics are shown in Table 1. The dropout group had a higher proportion of men and elder patients than the treatment completion group but without significance. There were more single patients, which included never married, divorced, or widowed, in the dropout group and significantly so. Tumor staged T4a accounted for more in the dropout group. Other baseline characteristics were not different.

**Table 1:** Baseline characteristics of patients with neoadjuvant chemotherapy for locally advanced oral cavity cancer

Characteristics	Undergoing surgery (*N* = 148)	Without surgery (*N* = 23)	Statistical value ^a^	*P*-value
Age, year			1.285	0.201
Mean ± SD	56.7 ± 10.8	59.8 ± 10.0		
Median (range)	59 (29–73)	63 (40–72)		
Gender, n			1.903	0.168
Male	109	20		
Female	39	3		
Marital status, n			5.056	0.025
Married	136	17		
Unmarried/ Divorce/ Widowed	12	6		
Smoking in men, n			2.045	0.153
Current smoker	85	12		
Former smoker/Nonsmoker	24	8		
Alcohol drinking in men, n			1.150	0.284
Light	68	10		
Heavy	41	10		
Educational level, n			0.025	0.874
College or above	49	8		
Below college	99	15		
Primary site, n			3.745	0.404
Tongue	63	14		
Bucca	30	2		
Gingiva	23	2		
Palate	4	1		
Floor of mouth	28	4		
HPV status, n			0.100	0.751
Negative	98	16		
Positive	50	7		
T classification, n			4.245	0.039
2/3	103	11		
4a	45	12		
N classification, n			1.330	0.539
0	32	3		
1	51	7		
2	65	13		
Clinical stage, n			1.485	0.223
III	65	7		
IVa	83	16		

*SD* standard deviation, *HPV* human papilloma virus

^a^ The Statistical values referred to the t-value for continuous variables and the Χ^2^-value for categorical variables

When combined in the regression model, variables of age over 65 years (OR = 3.110, 95%CI: 1.108, 8.733) and single marital status (OR = 4.935, 95%CI: 1.511, 16.121) were significant as a risk factor of dropout among the studied patients.

Table 2 shows the response and the adverse events of neoadjuvant chemotherapy in the two groups. Patients who dropped out after chemotherapy reported vomiting more severely. Response to chemotherapy and white blood cell decrease were not different between the groups.

**Table 2:** Response and adverse of neoadjuvant chemotherapy among patients with locally advanced oral cavity cancer

	Undergoing surgery (*N* = 148)	Without surgery (*N* = 23)	Χ^2^ value	*P* - value
Response ^a^			0.401	0.859
PR	81	14		
SD	60	8		
PD	7	1		
Adverse events ^b^				
WBC decreased			0.254	0.614
Grade 0/1	137	20		
Grade 2/3	11	3		
Vomiting			9.082	0.003
Grade 0/1	143	18		
Grade 2/3	5	5		

^a^ The response definitions were classified according to the Response Evaluation Criteria in Solid Tumors version 1.1

^b^ The adverse events were graded according to National Cancer Institute Common Terminology Criteria for Adverse Events version 5.0

### Follow-up information of the dropout patients

Among the 23 dropout patients, most had passed away when we telephoned. Only one patient had been alive for over 2 years when data censored. So, the information was mostly obtained from patients’ family members. The median survival time of the 22 patients were 7.4 months, ranging from 2 to 17 months. The reasons for patients refused to undergo surgery were summarized as: 1) symptoms relieved and thought that the chemotherapy would be effective, in 19 patients; 2) fearing of the consequences of surgery, that is the loss of oral function and unable to take care of themselves in daily life, in 14 patients; 3) be worried that no family members or relatives would look after them, in 7 patients. They originally wanted to have a radical curative treatment. But as time went on, when they felt symptoms relieved and saw the consequence of surgery of other patients, they changed their mind. They refused surgery and went on chemotherapy, chemoradiation, or other nonsurgical treatments, as suggested by their doctors. Five patients finally wanted to have surgery again, but lost the indications.

The living patient had a partial response to the TPF regimen. After refusal of surgery, he received another two cycles of medication followed by concurrent chemoradiotherapy and achieved complete remission.

## Discussion

There was no consensus on treatment modality for cancer patients who refused surgery after neoadjuvant chemotherapy. The characteristics of these patients, reasons for their decision of dropout, and their follow-up information were rarely reported in literatures. Clinical trials on cancer management were mostly for patients with primary tumor or metastatic/recurrent tumor. Before planning clinical trials for the dropout patients, we carried out this investigation.

### Nonsurgical treatment option

Most patients in the dropout group in our study believed that chemotherapy would be effective for their disease, because their symptoms relieved after using the medicine. Patients with oral cavity cancer were often scared of the radical performance because of the consequent loss of oral function and disfigurement of facial appearance. This fear of surgery may be more obvious in oral cancer patients than in patients with cancers of other sites. The TPF regimen had been reported a high response rate between 50 and 80% [4–8]. Though, these studies were conducted in preoperative medication, for the dropout patients, the relief of symptoms made them believed that the medicine would work for their disease and refused to accept the following surgery.

Surgical-based treatment is still the mainstay for locally advanced oral cavity cancer. However, the consequent loss of oral function is unavoidable and overall survival remained poor. Alternative therapies are appealed for by the patients who refused to undertake surgery.

Definitive chemoradiation has been long explored. Its overall survival rate has been reported from 15 to 63% [12–17]. The discrepancy in the overall survival rate may be resulted from the patient inclusion criteria and the use of different radiation techniques or chemo-medicines. Foster and his colleagues [18] reported a favorable outcome of definitive chemoradiotherapy with durable toxicity and considered it as a viable and feasible strategy for patients with locally advanced oral cavity cancer who did not want to undertake surgery.

There is a current interest in incorporating target medicines or immunotherapy in treatment for advanced oral cancers. The addition of cetuximab, an epidermal growth factor receptor monoclonal antibody, to the traditional platinum/5- fluorouracil regimen has shown a promising result [19, 20]. Its concomitant use with definitive radiotherapy also improved the locoregional control [21].

Another milestone of nonsurgical treatment option for oral cavity cancer is the advent of the immune modulating antibodies, such as programmed cell death protein-1 antibodies and its ligand [22, 23]. Though these studies are still pilot and inconclusive, the results are expected.

### Age and marital status in cancer treatment

Age and marital status may affect patients’ decision making in cancer treatment. Our study showed that patients with age over 65 years or with single marital status were at risk of abandon surgery after neoadjuvant chemotherapy. Elder patients may have more reasons than the young that made them unable to retain in the long-term treatment duration. Literatures have reported the reasons, such as comorbid conditions or progression of disease [24, 25]. However, be afraid of loss function and unable to take care of themselves were also the reasons for the elder to dropout. Among the 23 patients who did not complete the prescribed treatment, over half reported their worry of caring themselves after chemotherapy. Oral cavity cancer can have a negative impact on patients’ daily life. Chewing or swallowing becomes a problem that troubles the patients in feeding themselves. They may experience slurred speech and difficulties in communicating with others. Also the expected alteration of facial appearance will make them psychologically depressed [26–28]. In addition, chemotherapy is considered to be one of strong stressors to older patients [29, 30].

Marital status to the compliance of cancer treatment was inconclusive. However, single persons, which include never married, divorced, or widowed, had reported a poorer survival than the married ones [31–33]. One reason was that they were more likely to be insufficiently treated; while, married persons more frequently received definitive or potentially curative treatment [34–36]. This might be affected by social support, psychological problem, or economic aspects, which were partially mediated via marital status, on deciding the choice of cancer treatment modality. One study had showed higher proportion of receiving surgery for cancer in married persons than in the unmarried [37]. It was owing to the at-home day-to-day support that made the patients willing to take on the risks associated with surgery. In our study, the proportion of single person was higher in the dropout group. Their reasons of dropout included nobody to discuss with and lack of information on treatment.

### Communications between patients and clinicians

The main reason of withdrawn in this study may be the insufficient information patients obtained. From the telephone with family members, it indicated that patients believed chemotherapy being effective mostly because of symptom relief. At the same time, they saw that radical surgery would cause serious functional loss. However, sometimes patients’ understanding of disease was one-sided. Communication between patients and clinicians needs to be improved in clinical practice, including survival probability, toxicity of treatment, time to recurrence, treatment burden, and also the health-related quality of life, from the initial stage of consultation, treatment decision, management duration, to rehabilitation stage. It may also help patient understand the disease that let the patients access to other patients at the time of decision making. In any case, we should understand, accept, and respect the patients’ decision and do our best to support them.

There were some limitations of the study. First is its retrospective nature. The reasons of dropout were mainly based on recall of the patients’ families, because most of the dropout patients were no longer alive when we telephoned. It may create recollection bias. Other variables, such as depression, anxiety, distress, and personality, could affect the patients’ decision but had not been recorded in this study. There was a T2N1 patient in the study group which was not normally suited to neoadjuvant chemotherapy. The study had a small number of dropout patients, which is not strong enough for statistical power. However, this entity is not common and has little been mentioned previously. We presented the relative factors of absence of surgery and the follow-up information of these patients for further study.

## Conclusion

In our study, the survival of the dropout patients was poor except one still alive at last follow-up. Most patients died of dyspnea, tumor bleeding, aspiration, or systemic failure. Thus, the dropout patients formed a special entity and urged to be studied for. The main reasons of dropout were the thinking that the medicine would be effective for the disease and the fearing of the consequence of radical surgery. Clinical trials are needed to be designed for these patients who had undergone neoadjuvant chemotherapy but refused the following surgery.

## Data Availability

The data and materials that support the findings of this study are available from the corresponding author on reasonable request.
